# Supplementary material for: A cell-free nanobody engineering platform rapidly generates SARS-CoV-2 neutralizing nanobodies
Source: Nat Commun. 2021 Sep 17;12:5506. doi: 10.1038/s41467-021-25777-z (PMC8448731; doi:10.1038/s41467-021-25777-z)
Supplement: Supplementary file 1 — Supplementary Information [file 41467_2021_25777_MOESM1_ESM.pdf]

Supplementary Information

A cell-free nanobody engineering platform rapidly generates SARS-CoV-2 neutralizing nanobodies

Xun Chen[1,#], Matteo Gentili[2], Nir Hacohen[2,3,4], Aviv Regev[1,5,6,7,#]

[1]Klarman Cell Observatory, Broad Institute of MIT and Harvard, Cambridge, MA, USA
[2]Broad Institute of MIT and Harvard, Cambridge, MA, USA
[3]Department of Medicine, Harvard Medical School, Boston, MA, USA
[4]Center for Cancer Research, Massachusetts General Hospital, Boston, MA, USA
[5]Massachusetts Institute of Technology, Department of Biology, Cambridge, MA, USA
[6]Howard Hughes Medical Institute, Chevy Chase, MD, USA
[7]Current address: Genentech, 1 DNA Way, South San Francisco, CA, USA
[#] To whom correspondence should be addressed: xun@broadinstitute.org (X.C.), aviv.regev.sc@gmail.com (A.R.)

Supplementary Fig. 1

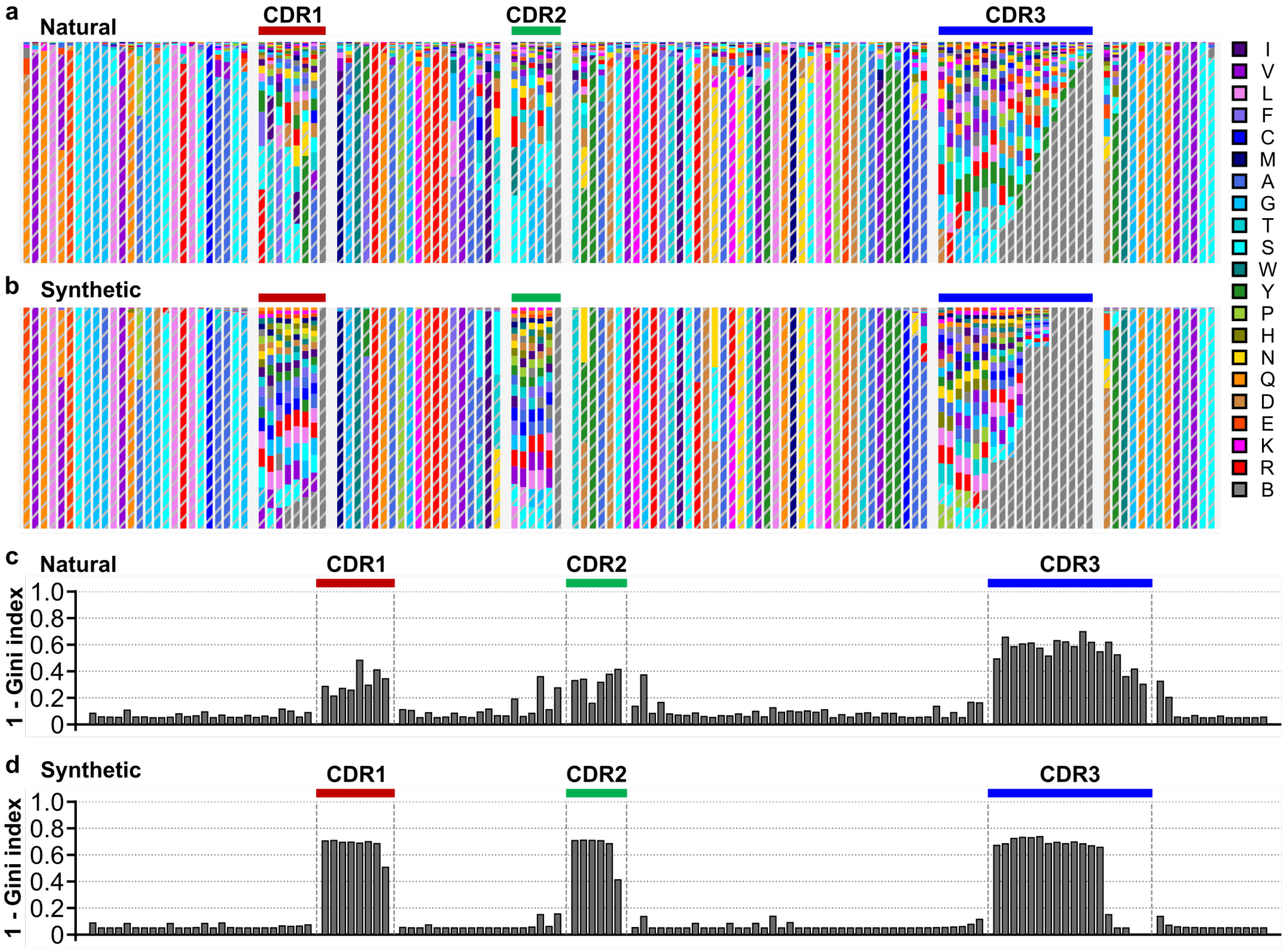

Supplementary Fig. 1. Amino acid profiles of natural and synthetic nanobodies. (**a**) Position-wise amino acid profile of natural nanobodies (298 nanobodies, PDB) and (**b**) synthetic nanobodies. Amino acids were color coded according to labels to the right, B indicates an empty position. Bar height: percentage of each amino acids. The two most common amino acids were shown as patterned bars while others were shown as solid bars. (**c**) Plot of diversity index (as 1 – Gini index) for each amino acid position of natural nanobodies and (**d**) synthetic nanobodies. Source data are provided in the Supplementary Data 2.

Supplementary Fig. 2

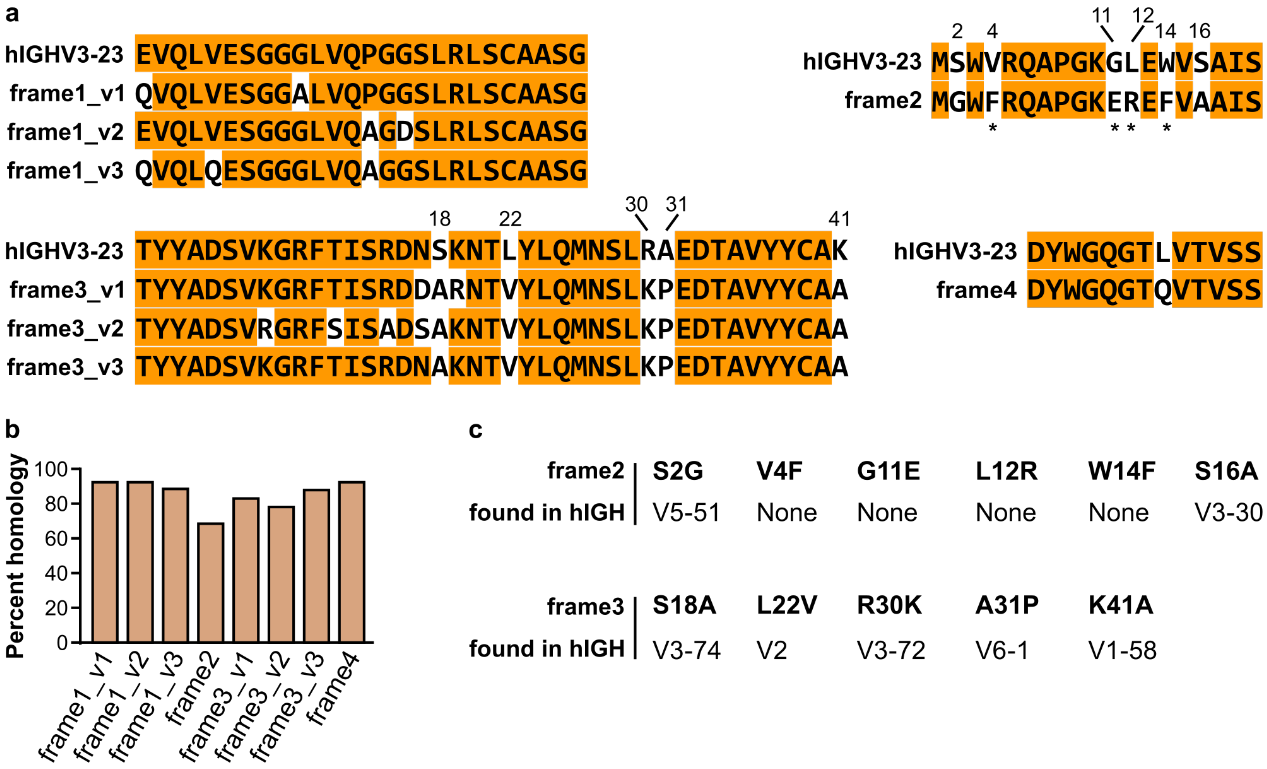

Supplementary Fig. 2. Design of nanobody frames and their homology to human IGH genes. (**a**) Amino acid sequences encoded by frames that serve as templates for nanobody library generation were aligned to the corresponding segments of the human IGHV3-23 (hIGHV3-23) or IGHJ4 (hIGHJ4). Positions in hIGHV3-23/hIGHJ4 that are identical to the corresponding position in at least one nanobody frames are highlighted in orange. Positions in nanobody frames that are identical to the corresponding position in hIGHV3-23/hIGHJ4 are highlighted in orange. hIGHV3-23 positions not identical to any nanobody frames are numbered according to its position within the segment. Asterisks indicate nanobody hallmark residues thought to be required for nanobody's independence of light chain. (**b**) Percent homology of nanobody frames to the closest human gene. (**c**) List of nanobody residues at positions numbered in (a) and representative human IGHV genes that encode the same nanobody residue at the corresponding position. None: no human IGHV genes has the nanobody residue at the corresponding position. Source data for (**b**) are provided in the Source Data file.

Supplementary Fig. 3

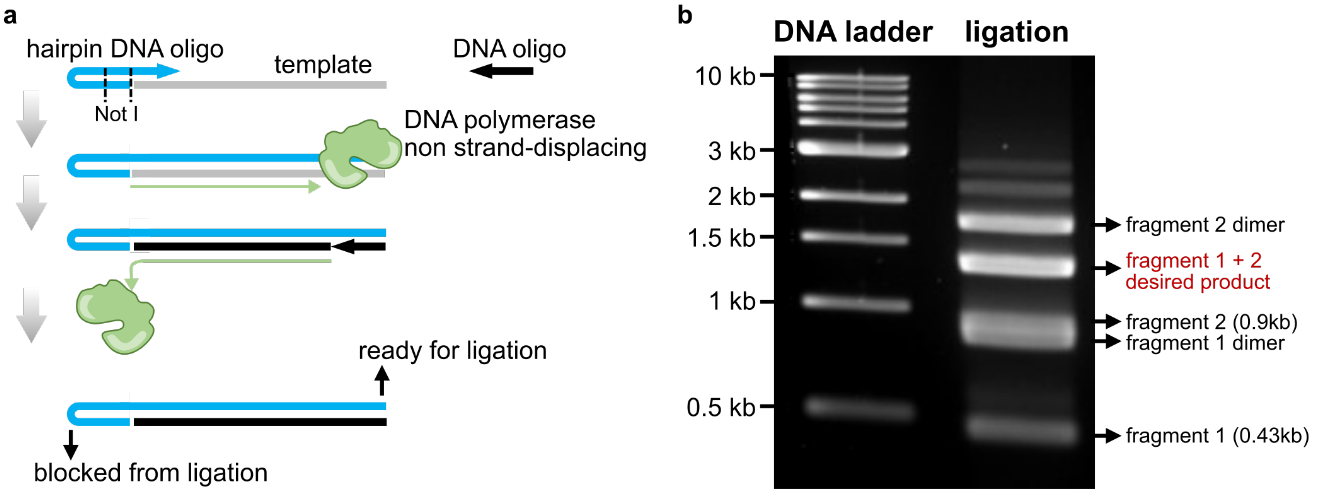

Supplementary Fig. 3. Working principle of orientation-controlled ligation by end blocking using hairpin oligos. (**a**) working principle for generating one end blocked DNA for orientation-controlled ligation by PCR using a hairpin DNA oligo. (**b**) Representative orientation-controlled ligation products visualized by agarose gel electrophoresis, representing five independent repeated experiments.

Supplementary Fig. 4

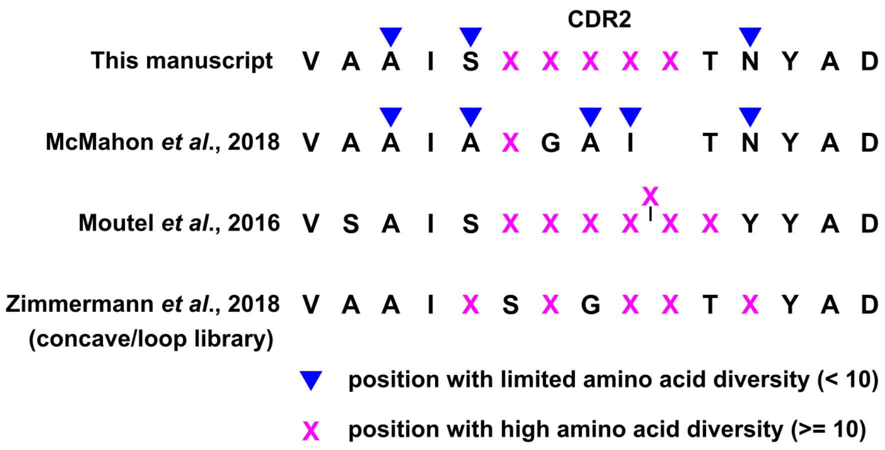

Supplementary Fig. 4. Comparison of the CDR2 region in the CeVICA nanobody library and previous designs. Alignment of CDR2 and neighboring sequences designed for four libraries (rows). X: highly diversified positions (>=10 different amino acids); blue triangles: positions with limited diversity (<10 different amino acids).

Supplementary Fig. 5

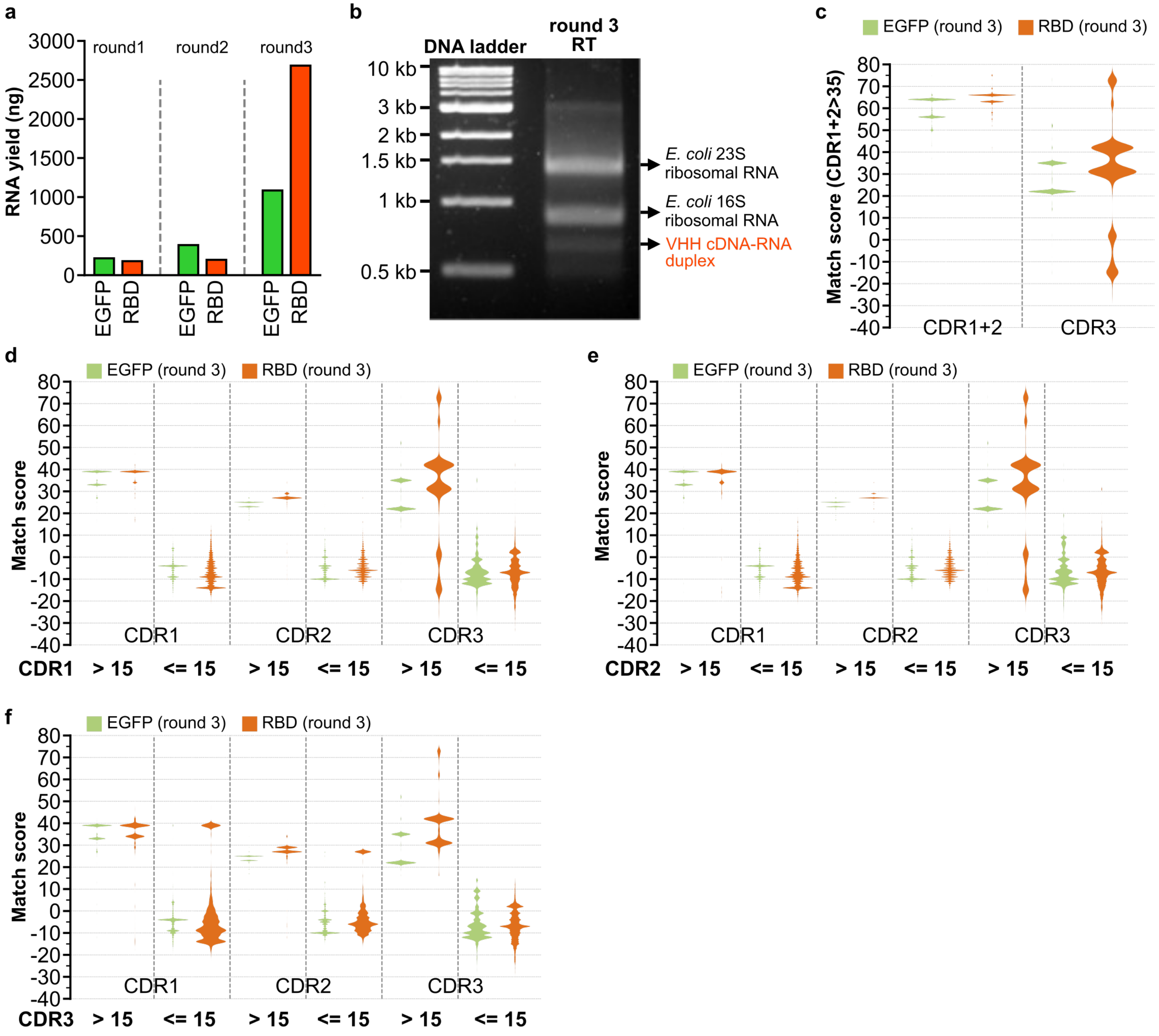

Supplementary Fig. 5. Evaluation of ribosome display and selection rounds. (**a**) Yield of recovered RNA at each round of ribosome display and selection for EGFP or RBD targets. (**b**) Representative RT reaction (without heat denaturation) product for RBD selection after 3 rounds, visualized by agarose gel electrophoresis, representing five independent repeated experiments. (**c**) Plot of match scores of sequence pairs with a combined CDR1 and CDR2 score > 35. (**d**) Plot of match scores of sequence pairs (from 2000 randomly sampled sequences) with indicated CDR1 scores, and (**e**) indicated CDR2 scores, and (**f**) indicated CDR3 scores. Source data for (**a**) are provided in the Source Data file.

Supplementary Fig. 6

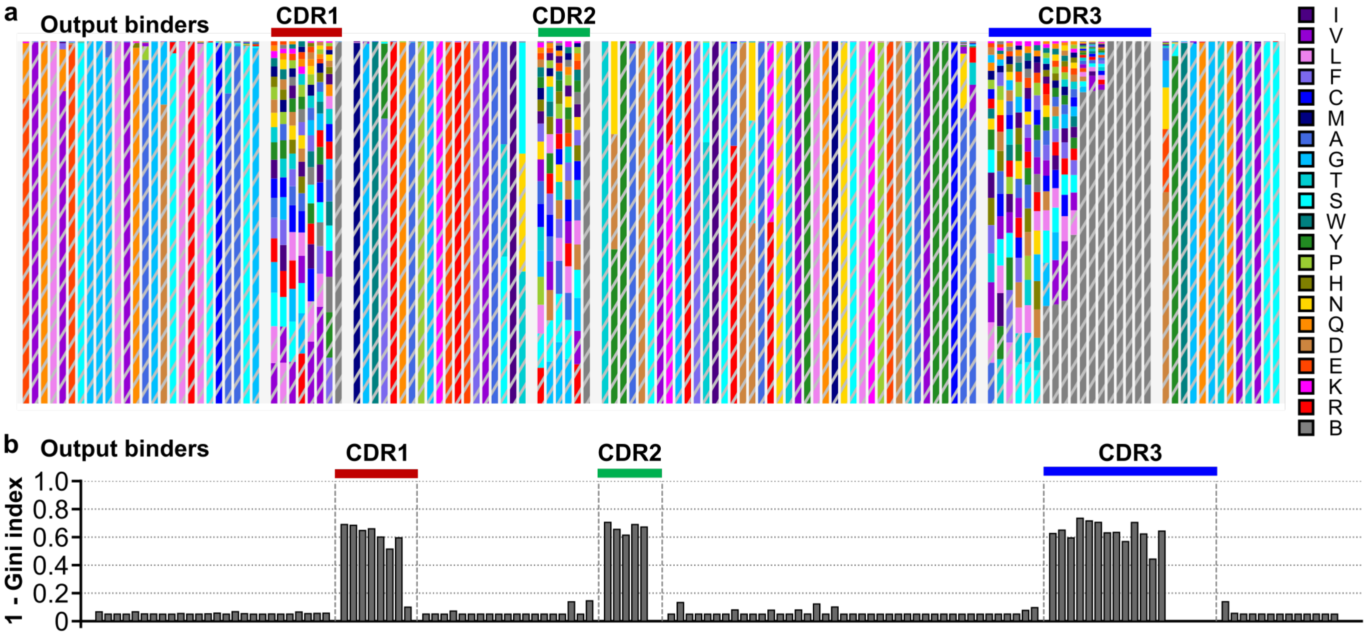

Supplementary Fig. 6. Amino acid profile for EGFP and RBD unique output binders. (**a**) Amino acid profile of representative nanobody sequence for each unique cluster identified from RBD and EGFP output libraries ("output binders", 932 sequences). Plotted as described in **Supplementary Fig 1a**. (**b**) Plot of diversity index (as 1 – Gini index) for each amino acid position of output binder nanobodies. Source data are provided in the Supplementary Data 2.

Supplementary Fig. 7

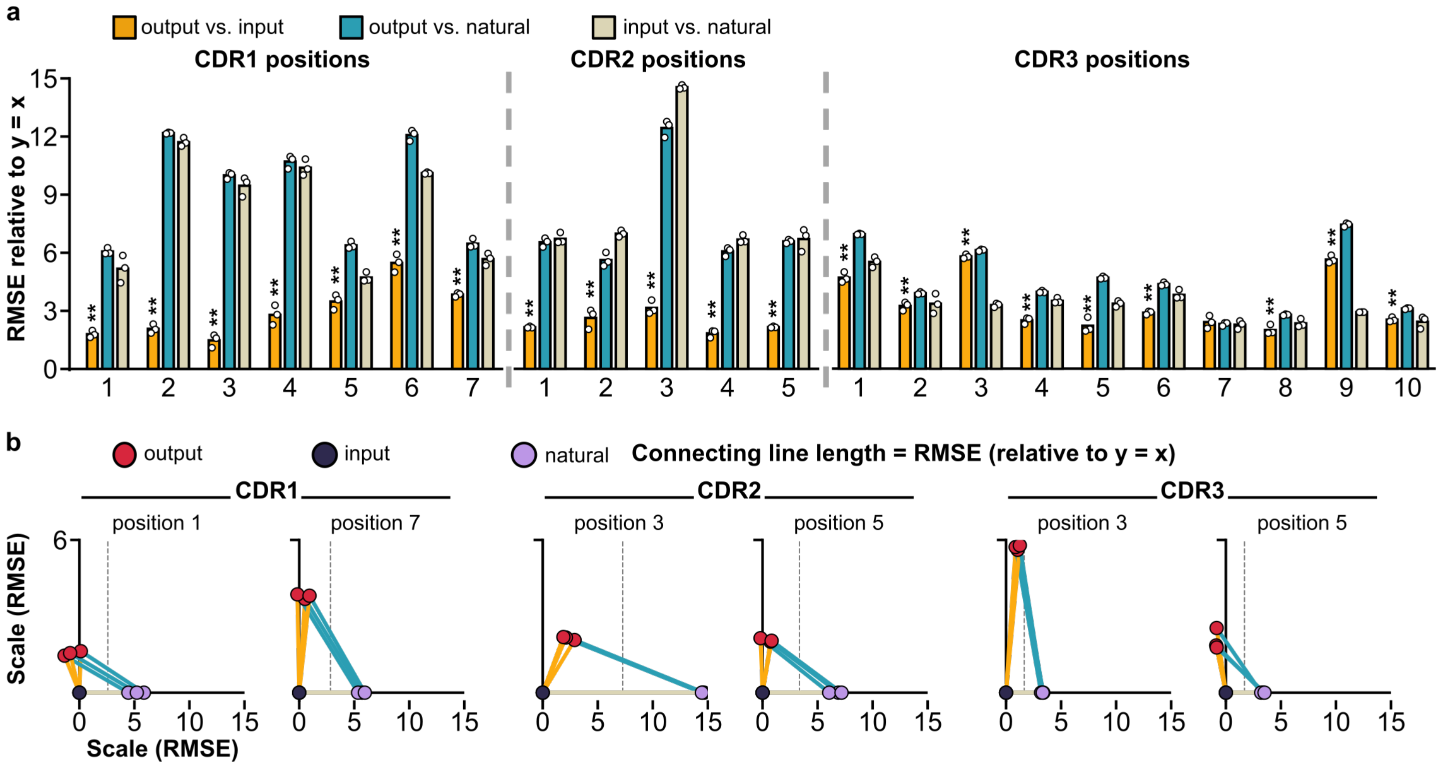

Supplementary Fig. 7. Comparison of CeVICA input and output amino acid profiles to that of 1,030 natural nanobody sequences in the abYsis collection. (**a**) Root mean square error (RMSE, relative to $y = x$ line) values for the amino acid percentages in 1,030 nanobodies from abYsis (natural), and 350 randomly sampled sequences from either the input (input) or output binders (output) libraries. Three random sampling trials were performed to generate three RMSE for each position. Bar height: mean, circle: value of each trial. **: $p < 0.01$, (two-sided t test between output *vs*. input and output *vs*. natural values, no multiple comparison adjustments). (**b**) Three-way distance maps of the distances between the three groups, with the length of each line connecting between two sequence groups indicating their RMSE. The input group (input) is fixed at (0,0), the natural group (natural) is fixed on the x axis (x,0), and the position of output group (output) is calculated based on its distance (RMSE) to the input and natural groups. Vertical dashed lines indicate the middle point of the distance between the input and natural groups. Source data are provided in the Source Data file.

Supplementary Fig. 8

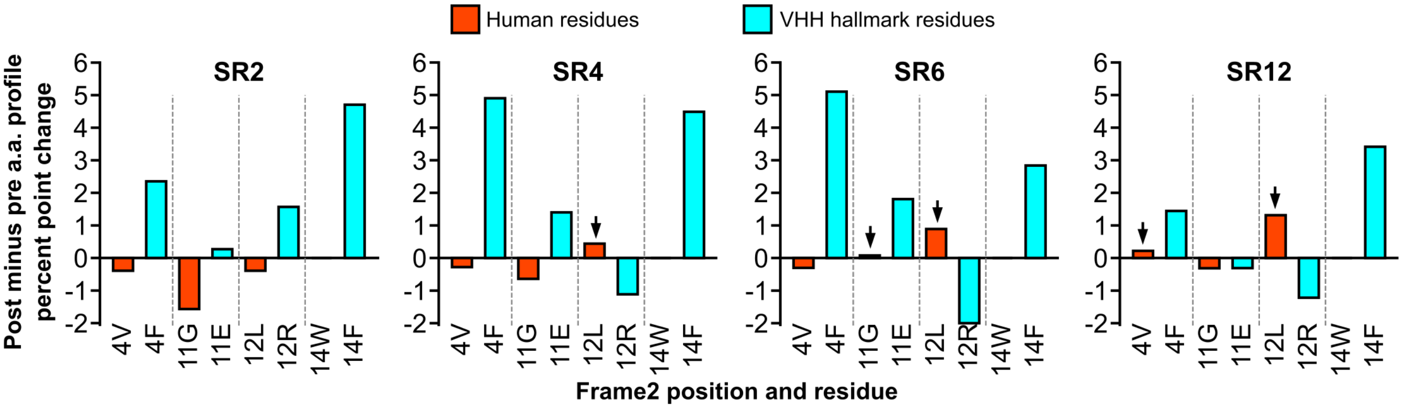

Supplementary Fig. 8. Affinity maturation leads to some VHH hallmark residues converting to the corresponding human VH residues. The post- minus pre-affinity maturation percent point change of VHH hallmark residues and the corresponding human residues for each nanobody. Arrows indicate human residues with increased frequency as a result of affinity maturation. Source data are provided in the Source Data file.

Supplementary Fig. 9

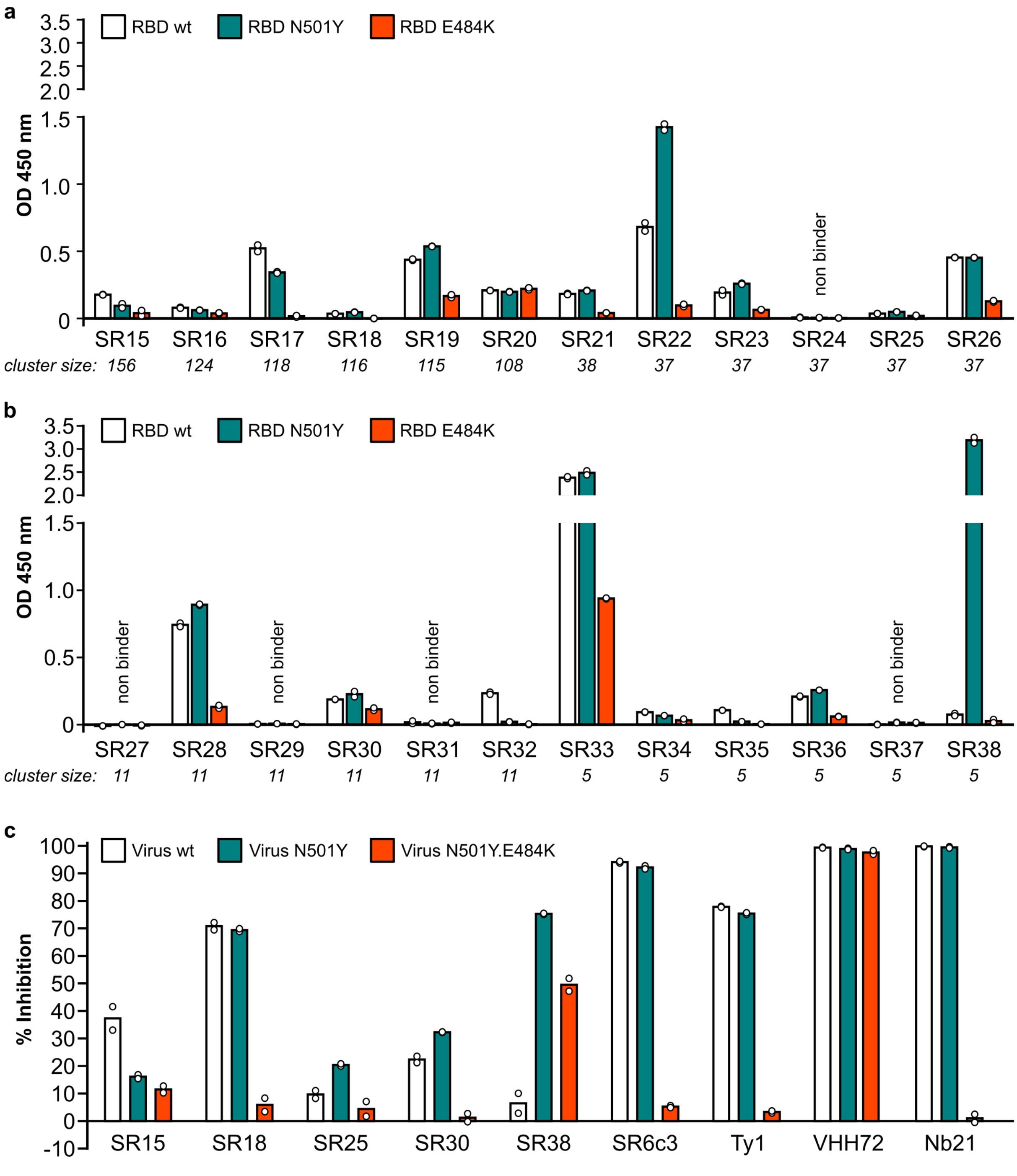

Supplementary Fig. 9. Identification of additional RBD binders and pseudovirus neutralizers among lower ranking clusters. (**a,b**) Binding assay. Binding measured by ELISA assay (y axis, OD 450 nm) of each of the nanobodies (x axis) tested at 1 µM for binding to wild type RBD (RBD wt) or RBD carrying the N501Y (RBD N501Y) or E484K (RBD E484K) mutations. Non binder: 5 nanobodies that showed undetectable values (background subtracted OD 450 nm < 0.02). (**c**) Pseudovirus neutralization. SARS-CoV-2 S pseudotyped lentivirus neutralization of nanobodies

at 1μM on HEK293T expressing ACE2 and TMPRSS2. Data shown are two technical replicates, bar height: mean, circle: value of each replicate. Source data are provided in the Source Data file.

Supplementary Fig. 10

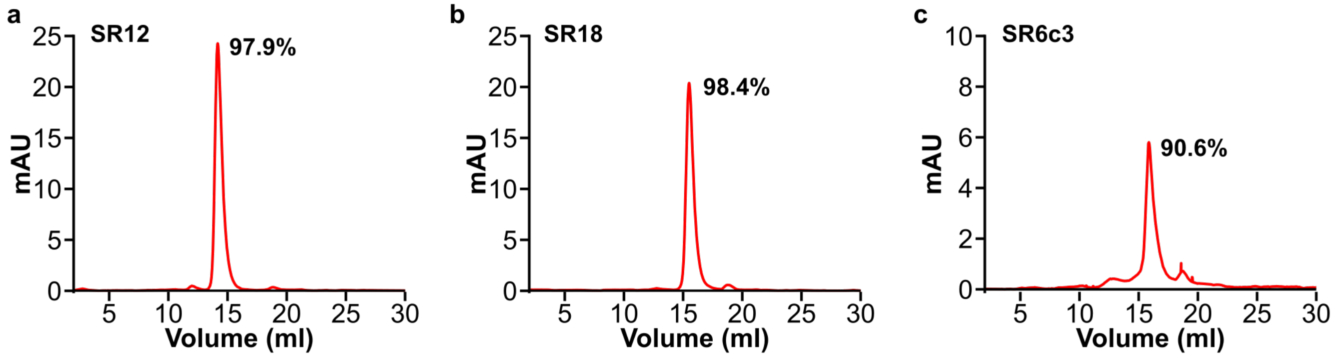

Supplementary Fig. 10. Representative nanobodies mainly exist as monomers in solution. Size-exclusion chromatography traces of SR12 (**a**), SR18 (**b**), and SR6c3 (**c**). Percent monomer values are shown next to the monomer peaks.

Supplementary Fig. 11

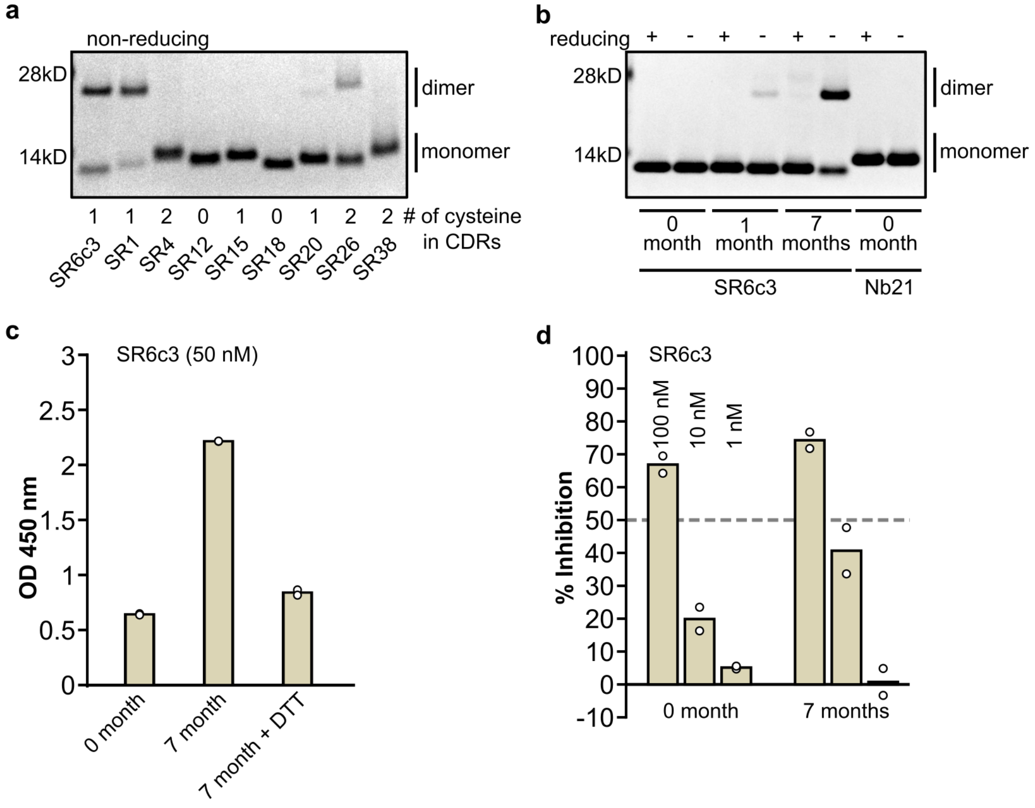

Supplementary Fig. 11. Intramolecular disulfide bond formation via CDR cysteine does not impair SR6c3 function. (**a**,**b**) Coomassie blue stained SDS PAGE gel of nanobody samples prepared in (**a**) non-reducing sample buffer, representing two independent repeated experiments, or (**b**) in non-reducing (-) or reducing (+) sample buffer, representing two independent repeated experiments. Number of months: length of time the sample has been stored at 4 °C. (**c**) Binding (y axis, by ELISA) of SR6c3 samples stored for different durations with or without treatment with DTT (50 mM DTT at room temperature for 2 hours). (**d**) SARS-CoV-2 S pseudotyped lentivirus neutralization of SR6c3 samples on HEK293T expressing ACE2 and TMPRSS2. Data shown are two technical replicates, bar height: mean, circle: value of each replicate. Source data are provided in the Source Data file.

Supplementary Fig. 12

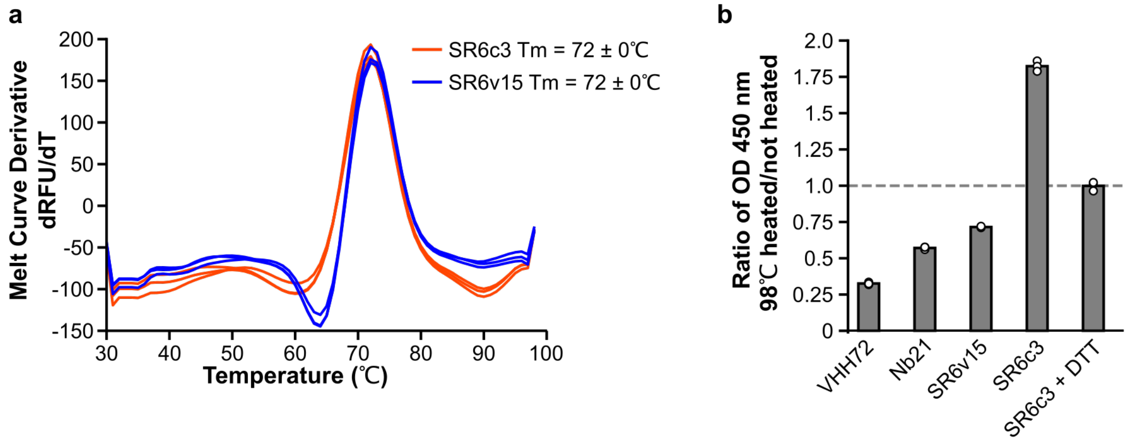

Supplementary Fig. 12. Thermal stability and refolding analysis of nanobodies. (**a**) Protein thermal shift assay. Melt curve derivative (y axis) at different temperatures (x axis) for two nanobodies. (**b**) Refolding assay following thermal denaturation. Ratio of binding (ELISA OD 450 nm) of heated (98 °C for 10 minutes) *vs.* non-heated samples (y axis) for different nanobodies (x axis). DTT: 25 mM DTT in sample solution. Data shown are three technical replicates, bar height: mean, circle: value of each replicate. Source data for (**b**) are provided in the Source Data file.
