# Supplementary material for: A cell-free nanobody engineering platform rapidly generates SARS-CoV-2 neutralizing nanobodies
Source: Nat Commun. 2021 Sep 17;12:5506. doi: 10.1038/s41467-021-25777-z (PMC8448731; doi:10.1038/s41467-021-25777-z)
Supplement: Supplementary file 12 — Description of Additional Supplementary Files [file 41467_2021_25777_MOESM12_ESM.pdf]

Title: Supplementary Data 1.
Description: Natural nanobody sequences selected for calculating natural nanobody amino acid profile. Amino acid sequences of 298 unique nanobodies selected from Protein Data Bank to represent natural nanobodies (PDB298, sheet: unique_VHH_PDB) and amino acid sequences of 1,030 unique nanobodies selected from abYsis to represent natural nanobodies (abYsis1030, sheet: unique_VHH_abYsis). The sequences were separated into 4 frames and 3 CDRs.

Title: Supplementary Data 2.
Description: Amino acid profile of the natural, input and output nanobodies. Position-wise amino acid profile of natural nanobodies, input library and output binder nanobodies from RBD and EGFP selection. Positions are relative positions within each segment and numbers are percentage of the corresponding amino acid labelled to the left of each segment.

Title: Supplementary Data 3.
Description: Primers and templates used for generation, selection and sequencing of nanobody library. Primer sequences, PCR cycling conditions and nanobody frame template sequences used in this study.

Title: Supplementary Data 4.
Description: List of RBD binder clusters. A list containing key information for all predicted RBD binding clusters (sheet: all clusters) and unique RBD binding clusters (sheet: all CDR unique). Cluster ID, size, CDR representative sequences, CDR consensus sequences, CDR scores (**Methods**), and whether each CDR is unique to RBD and not found in EGFP clusters were shown.

Title: Supplementary Data 5.
Description: List of EGFP binder clusters. A list containing key information for all predicted EGFP binding clusters (sheet: all clusters) and unique EGFP binding clusters (sheet: all CDR unique). Cluster ID, size, CDR representative sequences, CDR consensus sequences, CDR scores (**Methods**), and whether each CDR is unique to EGFP and not found in RBD clusters were shown. The cluster with ID 0, is a spike-in nanobody[16], and did not originate from the input library.

Title: Supplementary Data 6.
Description: Affinity maturation subtracted amino acid profile for SR4 and SR6. Position-wise post- minus pre- affinity maturation amino acid profile for SR4 and SR6. Numbers are percent point change of each amino acid after affinity maturation.

Title: Supplementary Data 7.
Description: Amino acid sequences of nanobody variant and the mutations they contain. Amino acid sequences of all nanobody variants characterized in this study.

Title: Supplementary Data 8.
Description: Nanobody variants ELISA and neutralization data. ELISA binding assay and pseudotyped virus neutralization assay results for all nanobody variants characterized in this study.

Title: Supplementary Data 9.
Description: High-throughput sequencing and analysis metadata. Number of sequences obtained by high-throughput sequencing for indicated analyses.

Title: Supplementary Data 10.
Description: Cluster files for SR1, SR2, SR4, SR6, SR8, SR12. Text file containing all sequences belonging to each cluster. Each line in the file represents one sequence, both segments and full length of the sequence were shown, shown items were divided by "#" and in the order from start to end of each line was: CDR1 amino acid sequence, CDR2 amino acid sequence, CDR3 amino acid sequence, full-length amino acid sequence, full-length DNA sequence.
