# Supplementary material for: A cell-free nanobody engineering platform rapidly generates SARS-CoV-2 neutralizing nanobodies
Source: Nat Commun. 2021 Sep 17;12:5506. doi: 10.1038/s41467-021-25777-z (PMC8448731; doi:10.1038/s41467-021-25777-z)
Supplement: Supplementary file 14 — Reporting Summary [file 41467_2021_25777_MOESM14_ESM.pdf]

nature research

Corresponding author(s): Xun Chen, Aviv Regev

Last updated by author(s): Aug 25, 2021

Reporting Summary

Nature Research wishes to improve the reproducibility of the work that we publish. This form provides structure for consistency and transparency in reporting. For further information on Nature Research policies, see our Editorial Policies and the Editorial Policy Checklist.

Statistics

For all statistical analyses, confirm that the following items are present in the figure legend, table legend, main text, or Methods section.

n/a	Confirmed	
☐	☒	The exact sample size (*n*) for each experimental group/condition, given as a discrete number and unit of measurement
☐	☒	A statement on whether measurements were taken from distinct samples or whether the same sample was measured repeatedly
☐	☒	The statistical test(s) used AND whether they are one- or two-sided<br>*Only common tests should be described solely by name; describe more complex techniques in the Methods section.*
☒	☐	A description of all covariates tested
☒	☐	A description of any assumptions or corrections, such as tests of normality and adjustment for multiple comparisons
☐	☒	A full description of the statistical parameters including central tendency (e.g. means) or other basic estimates (e.g. regression coefficient) AND variation (e.g. standard deviation) or associated estimates of uncertainty (e.g. confidence intervals)
☐	☒	For null hypothesis testing, the test statistic (e.g. *F*, *t*, *r*) with confidence intervals, effect sizes, degrees of freedom and *P* value noted<br>*Give P values as exact values whenever suitable.*
☒	☐	For Bayesian analysis, information on the choice of priors and Markov chain Monte Carlo settings
☒	☐	For hierarchical and complex designs, identification of the appropriate level for tests and full reporting of outcomes
☒	☐	Estimates of effect sizes (e.g. Cohen's *d*, Pearson's *r*), indicating how they were calculated

Our web collection on statistics for biologists contains articles on many of the points above.

Software and code

Policy information about availability of computer code

Data collection	SARS-CoV-2 S pseudotyped lentivirus neutralization assay data were collected using Cytoflex LX (Beckman Coulter) and data were analyzed with FlowJo v10.7. ELISA OD 450 nm measurement were performed using BioTek synergy H1 microplate reader with software Gen5 version 1.11.5. Size exclusion chromatography data were collected using the UNICORN 7 software (Cytiva). Biolayer interferometry data were collected using the Octet Data Acquisition Software 10.0 (Fortebio).
Data analysis	For VHH domain antibody sequencing and clustering analysis, custom python code was used and can be accessed through GitHub (DOI: 10.5281/zenodo.5257689). Data representation and standard statistical analysis were performed using the public python package Matplotlib 3.3.0 (https://matplotlib.org/). Size exclusion chromatography data were analyzed using the UNICORN 7 software (Cytiva). Biolayer interferometry data were analyzed using the Octet Data analysis software 10.0 (Fortebio).

For manuscripts utilizing custom algorithms or software that are central to the research but not yet described in published literature, software must be made available to editors and reviewers. We strongly encourage code deposition in a community repository (e.g. GitHub). See the Nature Research guidelines for submitting code & software for further information.

Data

Policy information about availability of data

All manuscripts must include a data availability statement. This statement should provide the following information, where applicable:

- Accession codes, unique identifiers, or web links for publicly available datasets
- A list of figures that have associated raw data
- A description of any restrictions on data availability

Antibody sequences are listed in Supplementary Data 7. Code for computational analysis are available on Github (DOI: 10.5281/zenodo.5257689). Key plasmids generated in this study will be deposited in Addgene. Natural VHH sequences were retrieved from Protein Data Bank (www.rcsb.org, date 2020-09-02,

Supplementary Data 1) and abYsis (www.abysis.org/abysis, date 2021-05-01, Supplementary Data 1)

Field-specific reporting

Please select the one below that is the best fit for your research. If you are not sure, read the appropriate sections before making your selection.

[x] Life sciences [ ] Behavioural & social sciences [ ] Ecological, evolutionary & environmental sciences

For a reference copy of the document with all sections, see nature.com/documents/nr-reporting-summary-flat.pdf

Life sciences study design

All studies must disclose on these points even when the disclosure is negative.

Sample size	Predetermined sample sizes were not calculated. Numbers of sequencing reads obtained were sufficient to produce the expected outcome that can be verified using independent assays. Experiments characterizing newly identified and engineered VHHs were performed with two or more independent replicates, which are sufficient given the large differences between different samples and controls. Sample sizes were chosen based on the reproducibility of each assay previously known by our experience and shown in related literature, the chosen sample size produced reproducible measurements that can be validated by other independent assays, for example, VHH samples showing positive ELISA binding measurement results also demonstrate inhibition of pseudovirus.
Data exclusions	Sequencing reads obtained from illumina Miseq platform were trimmed to remove bases with a quality score of less than 10, sequencing reads after quality score trimming that did not contain a full-length VHH sequence were discarded. This minimizes the influence of sequencing error. These exclusion criteria are determined prior to the experiments and analyses.
Replication	Number of repeats for each experiment are stated in figure legends. All repetition reproduced previous results. A previously characterized VHH domain antibody (VHH72) were included for characterization of new VHHs engineered in this study for both binding and neutralization.
Randomization	This study does not require allocation of animals/subjects.
Blinding	The investigator performing SARS-CoV-2 S pseudotyped lentivirus neutralization assay were blinded to the identity of test and control samples. ELISA and pseudotyped lentivirus neutralization assay both utilize quantitative measurement as readout with no manual scoring. For ELISA and biophysical characterization experiments on VHHs, investigators are not blinded to group allocations because these assays produce quantitative data measured by instruments that are not influenced by the investigators.

Reporting for specific materials, systems and methods

We require information from authors about some types of materials, experimental systems and methods used in many studies. Here, indicate whether each material, system or method listed is relevant to your study. If you are not sure if a list item applies to your research, read the appropriate section before selecting a response.

Materials & experimental systems

n/a	Involved in the study
[ ]	[x] Antibodies
[ ]	[x] Eukaryotic cell lines
[x]	[ ] Palaeontology and archaeology
[x]	[ ] Animals and other organisms
[x]	[ ] Human research participants
[x]	[ ] Clinical data
[x]	[ ] Dual use research of concern

Methods

n/a	Involved in the study
[x]	[ ] ChIP-seq
[x]	[ ] Flow cytometry
[x]	[ ] MRI-based neuroimaging

Antibodies

Antibodies used	monoclonal anti-Flag antibody (Sigma-Aldrich, Cat# F1804, clone M2), monoclonal anti-Myc antibody (ThermoFisher Scientific, Cat# 13-2500, clone 9E10), HRP conjugated anti-His tag secondary antibody (BioLegend, 652503, clone J099B12), newly engineered VHH in this study and the VHH72 were described in the paper.
Validation	Mouse anti-Flag antibody was validated by performing immuno-precipitation of EGFP with C-terminal 3XFlag tag and yielded a specific band corresponding to the correct sized of tagged EGFP in SDS-PAGE gels (data not shown). Mouse anti-Myc antibody was validated by pull down of ribosome display complex containing 3XMyc tag (Fig. 1h-j).

Eukaryotic cell lines

Policy information about cell lines

Cell line source(s)	The following cell lines were used in the study: HEK293T (ATCC CRL-3216), HEK293T ACE2 were a kind gift of Michael Farzan and originate from HEK293T (ATCC CRL-3216). HEK293T ACE2 cells were transduced with pTRIP-SFFV-Hygro-TMPRSS2 to obtain HEK293T ACE2/TMPRSS2 cells.
Authentication	HEK293T (ATCC CRL-3216) was confirmed to be a human cell line by sequencing multiple ribosomal protein genes from the cell line. HEK293T ACE2 and EK293T ACE2/TMPRSS2 cells were functionally verified to be susceptible to SARS-CoV-2 S pseudotyped lentivirus infection and are not authenticated by other methods.
Mycoplasma contamination	All cell lines were confirmed negative for Mycoplasma contamination monthly.
Commonly misidentified lines (See ICLAC register)	None
